# A set of prevention indicators for Bavaria – core data on prevention for Bavaria and its regions

**DOI:** 10.17886/RKI-GBE-2018-060

**Published:** 2018-05-16

**Authors:** Veronika Reisig, Joseph Kuhn

**Affiliations:** Bavarian Health and Food Safety Authority Department Health Reporting, Epidemiology, Social Medicine

In Bavaria systematic prevention reporting is being established. The aim is to support the implementation of the Bavarian Prevention Plan as well as of Bavaria's federal state framework agreement and to deliver regional data for national prevention reporting [1]. In a survey, more than 90 % of stakeholders in the Bavarian Prevention Alliance had asked for the provision of a core prevention data set [2].

Therefore, as one of the first products of Bavarian prevention reporting, such a data set was developed in 2017. The data set comprises a selection of important data relevant to prevention and health promotion. It is oriented along the action areas of the Bavarian Prevention Plan “Growing up healthy”, “Healthy adult and working life”, “Healthy aging” and “Health equity” [3]. In accordance with the targets of the Prevention Plan, indicators depicting the broader determinants of health (e.g. environmental or social aspects), health behaviour, health literacy, prevention activities and relevant outcomes were meant to be included for each action area. Important criteria for the selection of indicators were the availability of data for Bavaria and for Germany by gender and – if possible – by social status as well as the availability of regular data updates. Indicators referring to risks as well as to resources for health, behaviour and living conditions were included. Where available, references to small area data were given.

A working draft of the indicator set contains nearly 120 indicators. For about one third, small area data are available. Despite the abundance of prevention related data, data on risks outweigh by far those on resources, and there is a lack of systematically collected data on prevention activities. The Bavarian prevention stakeholders approved of the draft indicator set. It is meant to be published and updated regularly.
